# Semantic Segmentation of Sorghum Using Hyperspectral Data Identifies Genetic Associations

**DOI:** 10.34133/2020/4216373

**Published:** 2020-02-04

**Authors:** Chenyong Miao, Alejandro Pages, Zheng Xu, Eric Rodene, Jinliang Yang, James C. Schnable

**Affiliations:** ^1^Center for Plant Science Innovation, University of Nebraska-Lincoln, Lincoln, NE, USA; ^2^Department of Agronomy and Horticulture, University of Nebraska-Lincoln, Lincoln, NE, USA; ^3^Quantitative Life Sciences Initiative, University of Nebraska-Lincoln, Lincoln, NE, USA; ^4^Department of Mathematics and Statistics, Wright State University, Dayton, OH, USA

## Abstract

This study describes the evaluation of a range of approaches to semantic segmentation of hyperspectral images of sorghum plants, classifying each pixel as either nonplant or belonging to one of the three organ types (leaf, stalk, panicle). While many current methods for segmentation focus on separating plant pixels from background, organ-specific segmentation makes it feasible to measure a wider range of plant properties. Manually scored training data for a set of hyperspectral images collected from a sorghum association population was used to train and evaluate a set of supervised classification models. Many algorithms show acceptable accuracy for this classification task. Algorithms trained on sorghum data are able to accurately classify maize leaves and stalks, but fail to accurately classify maize reproductive organs which are not directly equivalent to sorghum panicles. Trait measurements extracted from semantic segmentation of sorghum organs can be used to identify both genes known to be controlling variation in a previously measured phenotypes (e.g., panicle size and plant height) as well as identify signals for genes controlling traits not previously quantified in this population (e.g., stalk/leaf ratio). Organ level semantic segmentation provides opportunities to identify genes controlling variation in a wide range of morphological phenotypes in sorghum, maize, and other related grain crops.

## 1. Introduction

A wide range of plant morphological traits are of interest and of use to plant breeders and plant biologists. The introgression of dwarfing genes which reduce stalk length and increase lodging resistance was a critical factor in the wheat cultivars that dramatically increased yields during the green revolution [1] as well as the widespread introduction of sorghum into mechanized agricultural production systems [2]. Increased yields in maize have largely come from selection for plants that tolerate and thrive at high planting densities [3], and modern hybrids have much more erect leaves than older hybrids, which have been shown to increase yield at high densities [3, 4]. Harvest index—the ratio of grain mass to total plant mass at harvest—is another critical plant property that has been a target of selection, either directly or inadvertently, in efforts to breed higher yielding and more resource use efficient crop varieties, particularly in wheat and barley [5]. Leaf initiation rate, leaf number at reproductive maturity, and the size and area of the largest leaf are all parameters employed in crop growth models to estimate plant performance in different environments [6]. These parameters are currently quantified using low-throughput and labor-intensive methodologies, limiting the feasibility of constructing models for large numbers of genotypes [7]. Semantic segmentation that distinguishes different plant organs increases the feasibility of computationally estimating many of the morphological parameters described here.

A number of straightforward thresholding metrics can be employed for whole plant segmentation, including excess green indices and image difference calculations using one photo with a plant and another otherwise identical photo without [8]. Nongreen plant organs such as mature seed heads can be identified against a background of leaves and stalks using deep learning methods, producing bounding boxes around the target organ [9]. Segmentation of leaves and stalks using 3D point clouds has been demonstrated in a range of crops including grape and sorghum [10, 11]. However, separating green stalks from green leaves in RGB images is a more challenging procedure. Hyperspectral imaging of plants has been successfully employed to both estimate plant nutrient status and to detect and classify disease identity, onset, and severity [12–16]. Plant organs have also been reported to exhibit distinct spectral signatures [17], including a difference in reflectance patterns between leaves and stems of maize plants for 1160 nm wavelength light [8]. These results suggest it may be possible to separate and classify plant organs based on distinct hyperspectral signatures.

Here, we explore the viability of using hyperspectral data to classify images of sorghum plants into separate organs with pixel level resolution. Using individual pixel labels generated using the crowdsourcing platform Zooniverse, leaves, stalks, and panicles is demonstrated to have distinct spectral signatures. A range of supervised classification algorithms are evaluated and a number of them provide high classification accuracy. We demonstrate that some of these organ level spectral signatures are conserved, as classifiers trained on sorghum data can also accurately classify maize stalks and leaves. Finally, organ level semantic segmentation data for a sorghum association population is employed to conduct several genome-wide association studies (GWAS). The identification of known genes controlling phenotypic variation for previously measured traits is recapitulated, and trait-associated SNPs are also identified for novel traits which can be quantified using the procedure described here. Overall, the data, methods, and pipeline introduced in the present study can aid further efforts to identify genes controlling variation in important morphological traits in both sorghum and other grain crop species.

## 2. Materials and Methods

**Figure 1:**
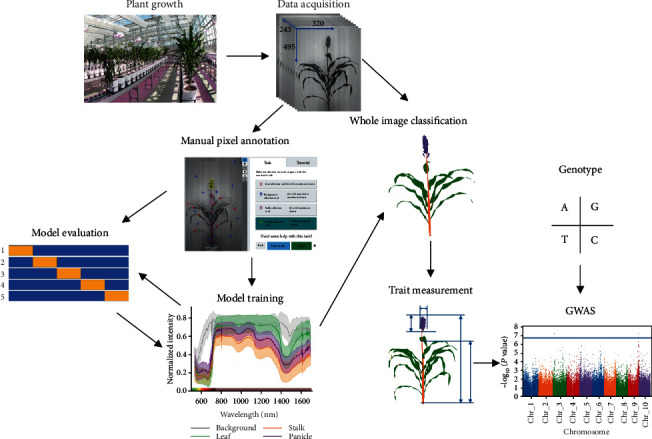
Steps involved in data acquisition, annotation, model training and evaluation, and genetic association analyses described in this study.

An overview of the experimental design and data flow for the analyses described in this manuscript is provided in Figure 1. The details of each stage of the process are described in a corresponding section of Materials and Methods below.

### 2.1. Plant Growth and Data Acquisition

A subset of 295 lines from the 377 line sorghum association panel (SAP) [18] were grown in the greenhouse of the University of Nebraska-Lincoln's Greenhouse Innovation Center (latitude: 40.83, longitude: -96.69) between June 14 and August 28, 2018. Forty days after planting (DAP), all plants were placed on previously described conveyor belt imaging and automatic watering system [8]. Each plant was imaged using a hyperspectral camera (Headwall Photonics, Fitchburg, MA, USA). Plants were arranged so that the axis of leaf phyllotaxy was as close to perpendicular with the line between the hyperspectral camera and the center of the stalk as possible. Hyperspectral images were captured at a resolution of 320 × 560 pixels. The camera employed has a spectral range of 546-1700 nm, with 243 distinct intensity values captured for each pixel (approximately 5 nm per band). At the zoom level used in this paper, for objects at the distance between the camera and plant, each pixel represents an area of approximately 3.1mm × 3.1mm (9.61 mm^2^). Maize plants used for the evaluation of model transferability were grown similarly in the same facility and imaged 66 DAP in September of 2018. Maize genotypes used for evaluation were drawn from the Buckler-Goodman 282 association panel [19].

### 2.2. Manual Pixel Annotation

A project—titled “Sorghum and Maize Segmentation using Hyperspectral Imagery”—was created on the Zooniverse crowdsourcing science platform (https://www.zooniverse.org/projects/alejandropages/sorghum-and-maize-segmentation-using-hyperspectral-imagery). Two different image datasets were uploaded to the project page for the pixel data annotation. The first dataset consisted of grayscale images of 189 sorghum plants at the grain fill stage of development. The second dataset consisted of 92 gray scale images of sorghum plants during the vegetative stage of their life cycle. For the first image dataset, users were directed to select ten pixels per class per image for four classes (background, leaf, stalk, and panicle) (Figure S1) and a total of 7560 classified pixels (189images × 4classes × 10pixels per class per image) were scored. Vegetative sorghum does not yet have a visible panicle. For vegetative stage sorghum plants, a total of 2760 pixels (920 per class) were scored. Based on timing classification speed, we estimate the marginal time required to classify each additional 1000 pixels to be approximately one hour. However, there are substantial fixed time costs to setting up and documenting each new experiment. These costs would be substantially greater if tools for farming out images and collecting annotations from workers are built from scratch rather than utilizing existing tools. The location of each pixel selected in Zooniverse was used to extract a vector of all 243 wavelength intensity values for that pixel from the original hyperspectral image cubes. The code used for converting raw Zooniverse classification output data to vectors of intensity values from the original hyperspectral images is provided as part of the GitHub repository associated with this paper.

### 2.3. Model Training and Model Evaluation

Seven supervised classification methods including multinomial logistic regression (MLR), support vector machine (SVM), linear discriminant analysis (LDA), partial least squares discriminant analysis (PLS-DA), random forest (RF), least absolute shrinkage and selection operator (LASSO), and quadratic discriminant analysis (QDA) were evaluated in R/3.51 using the data collected from grain fill stage sorghum as described above. The MLR classifier was trained using the “multinom” function provided by the “nnet” library with default parameter settings [20]. The SVM classifier was trained using the “svm” function provided by the “e1701” library with the parameter “probability” set to TRUE [21]. LDA and QDA classifiers were trained using “MASS::lda” and “MASS::qda” functions with the default parameters from “MASS” library [20]. The PLS-DA classifier was trained using the “plsda” function with the parameter ncomp = 10 from the “caret” library [22]. LASSO employed the “glmnet” function with parameter family = “multinomial” from the “glmnet” library [23]. The RF classifier was trained using “randomForest” function with the default parameters from the “randomForest” library [24]. The importance index for each feature was also estimated using “randomForest” function but with the parameter importance = TRUE.

A total of 600 artificial neural networks (ANNs) which varied in either architecture and/or hyperparameter settings were also evaluated. Each ANN was implemented in python/3.7 using Keras/2.2.4 library built on TensorFlow/1.11. A total of 15 different neural network architectures were tested, representing all possible combinations of three different numbers of hidden layers (2, 3, 4) and five different unit sizes for each hidden layer (200, 250, 300, 350, 400). For each architecture, 40 different learning rates sampled from a range between 1*e* − 3 and 1*e* − 6 using a uniform random distribution were tested. For all ANNs evaluated, the Relu activation function was employed on the hidden layers, Softmax on the output layer, and stochastic gradient descent (SGD) was employed as the optimizer during the training. Results from the single highest performing ANN—4 hidden layers, 300 units in each hidden layer, and a learning rate of 5.0*e* − 4—are presented. The corresponding R and Python code for all analyses is provided on GitHub (https://github.com/freemao/Sorghum_Semantic_Segmentation). Accuracy for all eight methods was evaluated using 5-fold cross validation to generate classifications for each observed pixel. Data was split into folds at the level of whole images, so that all pixels classified in an individual image were assigned to either the training or testing dataset. Accuracy was defined as the number of pixels assigned the same label by manual classifiers and the algorithm being evaluated divided by the total number of pixels classified by both manual classifiers and the algorithm. As this was a balanced dataset with four total classes, the null expectation for accuracy from an algorithm which assigned labels randomly is 0.25.

### 2.4. Whole Image Classification

Raw hyperspectral images were output by the imaging system as 243 grayscale images representing intensity values for each of the 243 separate wavebands. Each image was stacked together in a 3D numpy array (height, width, band) with each value representing the light reflectance intensity of a single pixel at a wavelength band with *x*- and *y*-axis position. The dimensions of the 3D numpy array were cropped to 319 × 449 (*x*dimension × *y*dimension) for sorghum and 239 × 410 for maize to exclude the pot and extraneous objects outside the background. The cropped 3D array was converted to a feature array of pixel vectors by flattening the *x* and *y* dimensions, yielding a 2D feature array of dimensions (*x* × *y*, number of bands). The resulting 2D array was then fed to the trained models for making predictions. The model output was a vector with length *x* × *y* representing the predictions for each pixel in the feature array encoded as either 0, 1, 2, or 3 representing background, leaf, stalk, and panicle, respectively. The vector was reshaped to the original dimensions, a 2D matrix with the dimensions (*x*, *y*). Finally, visualizations of the segmentation map were produced by converting each value in the 2D matrix to an RGB value where the value 0 for the background was converted to white (255, 255, 255), 1 for the leaves to green (127, 201, 127), 2 for the stalk to orange (253, 192, 134), and 3 for the panicle to purple (190, 173, 212).

### 2.5. Trait Measurement and GWAS

Based on the initial classification of images into four pixel categories, seven traits were quantified. Estimates of leaf, panicle, and stalk size were simply generated by counting the number of pixels assigned to each of these categories in each image. Leaf/panicle, leaf/stalk, and panicle/stalk ratios were calculated by simple division of the number of pixels observed for each class in each image. Height to top of panicle was calculated by taking the Euclidean distance between the stalk pixel with the smallest *y*-axis value and the panicle pixel with the greatest *y*-axis value (Figure S8). Genotypic data was taken from a previously published study which includes GBS-identified SNPs for the SAP population [25]. Of the 295 plants imaged in this study, 242 had published genotypic data. For GWAS (genome-wide association study), an additional 15 lines were excluded as manual examination of hyperspectral images indicated that they had not completed reproductive development by 76-77 DAP. The published SNP dataset was filtered to exclude SNPs with minor allele frequency (MAF) < 0.01, and a frequency of heterozygous calls >0.05 among the remaining set of 227 lines. A total of 170,321 SNP markers survived this filtering process and were employed for GWAS. Narrow-sense heritability for each trait was estimated as the proportion of phenotypic variation explained (PVE) as reported by Gemma/0.95 [26]. Each trait GWAS analysis was conducted using the FarmCPU/1.02 software with the parameters method.bin = “optimum”, bin.size = c(5e5,5e6,5e7), bin.selection = seq(10,100,10), and threshold.output = 1 [27]. Both population structure and kinship were controlled for in this analysis. The first five principal components of population structure were derived from the genotype data using Tassel/5.0 [28] and included as covariates in all GWAS analyses. The kinship relationship matrix for all lines phenotyped was estimated and controlled for as covariates within the FarmCPU software package [27]. The cutoff for statistical significance was set to achieve a Bonferroni corrected *P* value threshold of 0.05.

## 3. Results

### 3.1. Hyperspectral Signatures of Sorghum Organs

**Figure 2:**
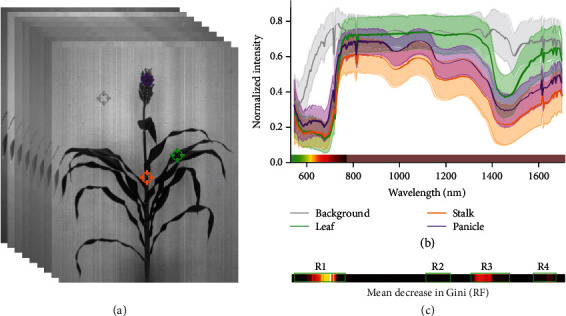
Distinct reflectance patterns of manually classified hyperspectral pixels. (a) A representation of a hyperspectral data cube with 254 image bands from 546 nm to 1700 nm. Example background, leaf, stalk, and panicle points highlighted in gray, green, orange, and purple, respectively. (b) Generalized reflectance patterns of leaf, stalk, panicle, and background pixels across wavelengths. Average reflectance intensity at each wavelength is indicated with a solid line, while the standard deviation among pixels belonging to that class is indicated by semitransparent bands. The blue portion of the visible spectrum 380-545 nm was not captured by this particular hyperspectral camera. The remaining portion of visible spectrum 546-780 nm or approximately green to red is indicated immediately above the *x*-axis. Infrared 780-1700 is indicated in the same color bar as pale brown. (c) Estimated feature importance for individual hyperspectral bands in random forest models indicated using the same *x*-axis scale of wavelengths used in (b).

Data extracted from a total of 7560 pixels from 189 images manually classified into one of four classes (background, leaf, stalk, and panicle) (Figure 2(a)) was used to plot average reflectance pattern for pixels assigned to each of the four classes (Figure 2(b)). Stalk and leaf exhibited very similar patterns in the visible portion of the spectrum, but clearly distinct patterns of reflectance in infrared. Stalk and panicle exhibited similar trends in infrared range from 750 nm to 1700 nm. Approximately 90% of total variance among manually classified pixels could be explained by the first two principle components of variation. Leaf and background pixels were clearly separated by these first two PCs; however, stalk and panicle pixels had overlapping distributions (Figure S2A). A similar pattern, with even less differentiation of stalk and panicle pixels, was observed for linear discriminant analysis (Figure S2B).

### 3.2. Performance of Classification Algorithms

A set of 8 supervised classification algorithms were evaluated for their ability to correctly classify hyperspectral pixels (Table 1). The average classification accuracy of five algorithms—estimated from fivefold cross validation—exceeded 96%. LDA achieved the highest overall prediction accuracy of >97%. As expected, given the distinct reflectance patterns observed in (Figure 2(b)), all the methods have very high accuracy on the classification of background pixels, and all methods also exhibited quite high (>96%) accuracy for leaf pixels. SVM, LDA, and PLS-DA had the highest accuracy for leaf (97.8%), stalk (94.6%), and panicle (97.6%), respectively, although the overall differences were quite small.

Mean decrease in Gini was calculated for the random forest model to identify those regions of the spectral curve which played a larger role in distinguishing between different classes. Spectral regions with a mean decrease in Gini > 10 were detected (Figure 2(c)). The first region (R1) is within the visible spectrum from 599 nm to 789 nm. This region may be capturing visible color differences between panicle and leaf/stalk, as well as visible light differences between background pixels and all three plant organs. R2 (1123-1218 nm) is in the near infrared and encompasses 1160 nm, a wavelength previously identified as useful for distinguishing leaves and stalks in hyperspectral images of corn [8]. R3 (1304-1466 nm) captures a local peak of water absorption. All three plant organs have significant water content and the background does not; this is a region that shows substantial differences between plant and nonplant reflectance spectra. The final region containing multiple spectral bands with mean decrease in Gini > 10 is (R4) is located between 1576 and 1652 nm.

**Figure 3:**
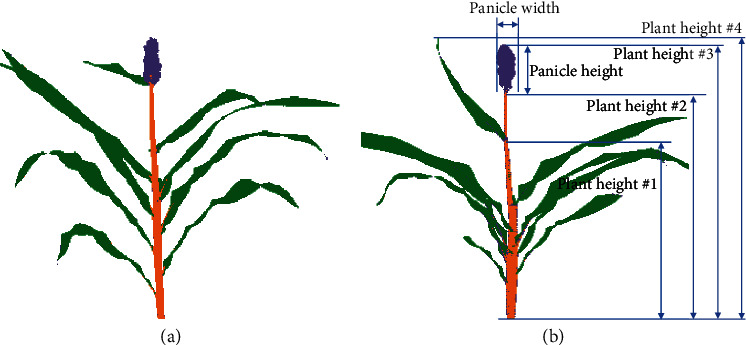
Whole image semantic segmentation of sorghum plants. (a) An example of a single sorghum plant with each pixel classified as either background (white), leaf (green), stalk (orange), or panicle (purple) using the LDA classifier described in Table 1. (b) Examples of a number of morphological traits which may be estimated using a semantically segmented sorghum image. Examples of four different definitions of plant height used by different researchers are indicated as follows: #1: height to flag leaf collar, #2: stalk height, #3: height to apex, and #4: height to the tallest point.

Each hyperspectral image collected as part of this study includes 179,200 pixels. Estimates of accuracy described above are based on manual annotation of individual pixels. However, as annotators were able to decide which 40 pixels to classify in a given image, manually annotated training data may exhibit a bias towards easy to visually classify pixels. Semantic segmentation was performed for a whole image using LDA (Figure 3(a)), the best-performing algorithm identified in Table 1. Qualitatively, classification accuracy appeared high. The most common error was small patches of pixels in the center of leaves which were misclassified as stalk. The thick, lignified midribs of sorghum leaves may produce reflectance patterns with greater similarity to stalk tissue than to the remainder of the leaf blade. The pixel level semantic segmentation of sorghum hyperspectral images enables the automated estimation of a range of plant traits. A notable example is that the simple trait “plant height” can correspond to at least four differently defined measurements collected by different plant breeders and plant biologists:

- Height to the flag leaf collar. Here, plant height is defined as the distance between the ground and the point at which the upper most—and last initiated—plant leaf joins the stalk
- Stalk height. Here, plant height is defined as the distance between the ground and the highest point on the stem
- Height to apex. Here, plant height is defined as the distance between the ground and the highest point on the stem or inflorescence
- Height to tallest point. Here, plant height is defined as the distance between the ground and the absolute highest point on the plant, frequently a leaf tip, but on other plants the highest point on the inflorescence

As illustrated in Figure 3(b), each of these definitions can produce a different measurement of plant height and all three can be estimated from images of sorghum plants classified into three organ types, while only the fourth definition of plant height is straightforwardly estimated from whole plant segmentation data.

### 3.3. Sorghum Model Transferability to Maize

Subsampling to create training and testing datasets can lead to over estimates of prediction accuracy in real-world use cases where more differences are likely to exist between training and application datasets. To evaluate the transferability of the trained models described above, a second dataset consisting of hyperspectral images of maize plants was employed. Maize and sorghum are related species within the tribe Andropogoneae. Both species have similar vegetative—leaf and stalk—architectures. However, the inflorescences of the two species are quite different. Both maize and sorghum datasets were collected using the same greenhouse imaging system, but data was collected at different times with different zoom levels. A set of 4000 pixels were manually annotated for background, leaf, stalk, and tassel classes.

**Figure 4:**
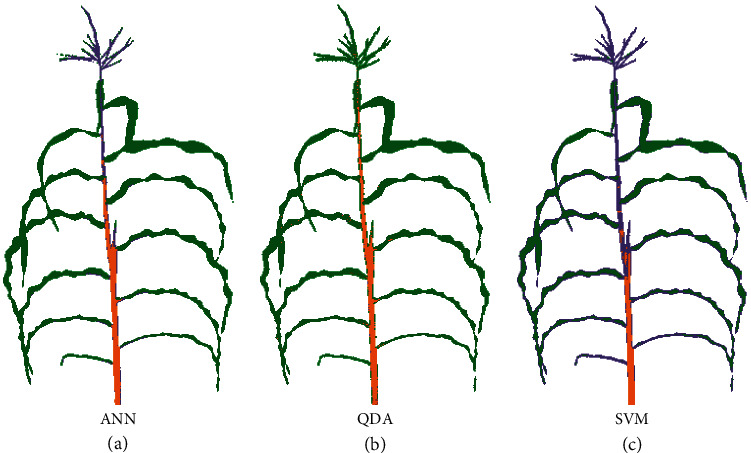
Example outcomes when classifying maize images using models trained on sorghum. (a) Whole image segmentation of a maize plant at flowering—genotype A635—using the best-performing sorghum-trained ANN as determined by cross validation accuracy in sorghum. Pixels predicted by the model to be background, leaf, stalk, and panicle are indicated in white, green, orange, and purple. (b) Whole image segmentation of the same maize plant by a QDA model trained on sorghum data. (c) Whole image segmentation of the same maize plant by a SVM model trained on sorghum data.

This 4000 pixel dataset was used to evaluate the overall and class-specific performance of each model trained on sorghum data in classifying pixels from the maize dataset (Table 2). As expected, cross-species prediction accuracy was lower than accuracy observed from cross validation within the sorghum dataset. Declines in accuracy were lower for background and leaf classes. Six out of eight models have a better performance in stalk than tassel. Low tassel/panicle accuracy in particular was expected as there are many differences between these two organs. The tassel is one of two specialized inflorescence types in maize. Unlike the sorghum panicle, the tassel is a specialized male reproductive structure and does not produce seeds. While LDA, ANN, and MLR all performed quite well on sorghum cross validation, LDA and MLR both dropped off significantly when sorghum-trained models were used to classify pixels from the maize dataset. Poor-performing classification models failed in a number of ways including misclassification of many tassel pixels as leaf (QDA) and misclassification of many stalk pixels as panicle (SVM) (Figures 4 and S4). ANN provided the best classification accuracy in maize of any of the sorghum-trained models (Figure S3). The gap in classification performance between ANN and the next best-performing model was greater when the partially nonequivalent tassel/panicle class was excluded.

### 3.4. Quantitative Genetics of Semantic Segmentation Traits

**Figure 5:**
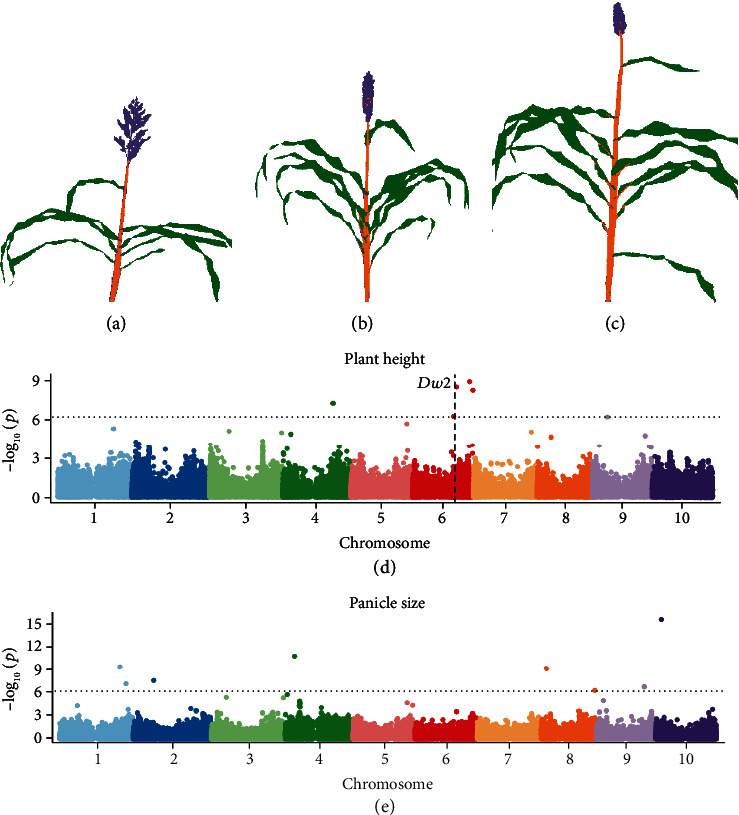
Mapping genomic regions controlling variation in sorghum phenotypes. (a–c) Examples of LDA-segmented sorghum plant images with short (a), medium (b), and tall (c) heights to apex and small (c), medium (b), and large (a) panicle sizes. (d) Results from a genome-wide association study for plant height to apex measured using results from LDA segmentation of images of 227 sorghum plants. The horizontal dashed line indicates a Bonferroni multiple testing-corrected threshold for statistical significance equivalent to *P* = 0.05. The vertical dashed line indicates the genomic location of *dwarf2*, a gene known to control variation in plant height in sorghum. (e) Results from a genome-wide association study for panicle size measured using results from LDA segmentation of images of 227 sorghum plants.

A key reason to produce pixel level organ classifications is that these make it easier to automatically quantify a range of plant phenotypes (Figure 3(b)). In many use cases in plant genetics and plant breeding, phenotypes where variance is primarily controlled by genetic factors will be of greatest interest. In others, phenotypes which are predominantly responsive to environmental factors will be of greatest interest. Phenotypes which vary a great deal from one plant to the next in patterns controlled by neither genetic nor environmental factors will be harder to study, and in some cases can be a sign of high error rate in measurement. Seven phenotypes were quantified from the whole image segmentation results from 227 sorghum plants each representing a distinct genotype from the sorghum association panel [18] (Figure S8). Three of these phenotypes were simple counts of pixels assigned to each of the three organ classes, stalk, leaf, and panicle. Three additional phenotypes were determined based on the ratios between these three classes. Finally, plant height to apex, one of at least four potential definitions of plant height, and a value difficult to calculate from purely plant/nonplant segmentation, was calculated for each plant. Narrow sense heritability—the proportion of total variance attributable to additive genetic effects—was estimated for each of the seven traits, using previously published SNP data for these 227 sorghum varieties (Table S1). Panicle size and plant height both exhibited significant phenotypic variation in the population of sorghum plants imaged (Figure 5(a)–(c)), as well as high estimated narrow sense heritabilities in this population (0.85 and 0.63, respectively). Estimated narrow sense heritability for leaf size was intermediate (0.32) and for stalk size was quite low (0.18). As stalk/leaf and panicle/stalk ratios both incorporated a very low heritability trait, the heritabilities for these traits were also low, while the estimated heritability of panicle/leaf ratio was higher (0.62).

Simple GWAS tests were also conducted for each trait (Figure 5(d) and (e), S5). It must be noted that this was a small population and does not include replication; however, at least one statistically significant trait-associated SNP was identified for each of the four traits with the highest estimated narrow sense heritability: plant height to apex, panicle size, panicle/leaf ratio, and panicle/stalk ratio (Table S1). In many cases, the genes and regulatory pathways controlling these genes have not been closely studied in sorghum previously. However, several of the associations we identify are consistent with reports from previous association studies in sorghum using other phenotyping approaches. The single SNP most significantly linked to variation in panicle size was located on chromosome 10, which was close to a locus identified in a previous study of panicle area and panicle solidity based on RGB images [29]. Individuals carrying the minor allele for this SNP frequently had open and diffuse panicle structures, as well as producing additional inflorescences from axillary tillers/branches (Figure S6). The significant SNP identified on chromosome 8 for the panicle size here is also adjacent to the locus which showed a significant association with multiple panicle solidity traits in the RGB study [29]. Plant height to apex has been the subject of intensive breeding efforts and genetic investigation in sorghum, and one trait-associated SNP in the GWAS for plant height to apex was located 33 kilobases away from the characterized *dwarf2* (*dw2*) gene on chromosome 6 [30, 31]. The two significant SNPs at the end of chromosome 6 are close to a locus for sorghum height identified in a separate sorghum MAGIC population (QTL-6) [32]. Each trait-associated SNP and annotated genes located within a 50 kb window up and downstream from each trait-associated SNP for each GWAS analysis shown in Figure 5(d) and (e) and S5 are provided in Additional file S1. The window size of 50 kb was selected as linkage disequilibrium decays below 0.2 at this distance in the SAP [25].

## 4. Discussion

### 4.1. Contribution

In this study, a set of hyperspectral images for both sorghum and maize association populations were generated using a high-throughput phenotyping platform. Each hyperspectral cube contains 254 band channels from 546 nm to 1700 nm covering part visible and infrared spectrums. A total of 7650 pixels from sorghum images including background, panicle, leaf, and stalk classes were manually annotated using the Zooniverse crowdsourcing platform, which substantially reduced the amount of tool development necessary to able to record both the locations of the clicked pixels and the corresponding label information in order to generate ground truth data. Eight machine learning algorithms were evaluated through the fivefold cross validation and the majority of them showed good performance on the sorghum semantic segmentation task. To test whether calculated accuracies were unrealistically optimistic as a result of classifiers selecting “easy pixels,” whole image predictions were also assessed qualitatively and accuracy appeared good. However, it should be noted that, as pixel level whole image manual classification was not conducted, so assessments of whole images represent a qualitative rather than quantitative metric. The feasibility of using trained sorghum models on the maize hyperspectral cubes were also tested. Although the plant pixels can still be clearly separated from the background, misclassifications of plant pixels as belonging to unexpected organs were more common. This was especially true for the tassel, likely as a result of the substantial biological differences between the sorghum panicle and maize tassel. Finally, traits extracted from sorghum data were shown to be under genetic control through estimates of narrow sense heritability, and it proved possible to identify genetic markers associated with variation in traits with high estimated narrow sense heritability, including one marker tagging a gene known to play a large role in controlling variation for the analyzed trait (*dw2*). These results demonstrate the potential of pixel level classifications of individual plant organs to automate the measurement of a range of morphological traits, assisting future plant genetics and plant breeding efforts.

### 4.2. Limitations and Future Work

The work presented above represents a proof of concept that hyperspectral imaging data can enable accurate organ level semantic segmentation of crop plants. However, there are several remaining challenges which should be addressed in order for this approach to have significant impact in the fields of plant biology and plant breeding. The first challenge is ensuring the accuracy of organ level pixel classifications across more diverse datasets. Classification accuracy was quite high when both training and testing on sorghum data collected at the same time, and lower with training on sorghum data collected at one time and testing on maize data at another. We also tested model generalizability within the sorghum, assessing the accuracy of models trained on grain fill stage sorghum on a separate set of images collected at the vegetative stage. Prediction accuracy declines somewhat from cross validation accuracy, indicating some degree of overfitting, but remains quite high (>95%) and higher than generalizability to maize, even for equivalent organs (e.g., leaf and stalk) (Table S2, Figure S7). While overfitting is a common phenomena when training and making predictions on distinct datasets, the average accuracy of SVM and LDA models is still over 95%, suggesting semantic segmentation approaches can be applied across different sorghum datasets. Generalizability could be tested further in the future using data from sorghum plants grown in different environments and subjected to different stresses.

Future work could seek to improve the robustness of prediction models through the collection of manual data from a wider range of experiments. Another potential avenue for improvement would be to incorporate either local or global spatial information into predictions. The algorithms tested in this study perform classification based on the hyperspectral signatures of individual pixels without considering data from neighboring pixels or position within the image. Postprocessing can reduce noise using methods such as median blur, erosion, or dilation approaches [33, 34]. Alternatively, directly incorporating intensity values from neighboring pixels when training pixel level classificaiton has been shown to improve whole plant segmentation accuracy for RGB images [35]. More complicated models considering the spatial architectures in the image, such as CNN, could also be applied on the segmented images either to improve segmentation accuracy or to extract higher level traits such as the number of leaves or to locating the position of each leaf [9, 36].

The phenotypes extracted in this study are arithmetic combinations of pixels which can cover a lot of traditional traits such as plant height and organ sizes. However, there are many more biologically relevant traits it may be possible to explore using these semantically segmented images. For example, the number of leaves and the flowering status can be obtained using CNN regression and classification models of RGB images [36, 37] but accuracy may be improved using images which are already semantically segmented. Plant architecture-related traits such as leaf curvature and stem angle can be estimated using more complicated mathematical algorithms [38]. In contrast, some traits can be only extracted or are much easier to be extracted from semantic images than the normal RGB images such as the phenotypes we present in this study. One simple to measure trait from semantically segmented images which was not assessed in this study was stalk width. Hyperspectral cameras sacrifice spatial resolution for spectral resolution and sorghum stalks in the images collected as part of this study were approximately 5-6 pixels wide. Higher zoom levels would enable more accurate quantification of stalk width with the same camera, but in this case, many other portions of the sorghum plant would not be included in the image. A key risk of using metrics estimated from 2D images is that, although we tried to adjust each plant so that the axis of leaf phyllotaxy was perpendicular to the camera, sorghum plants are not perfectly bilaterally symmetric and bias and error from viewing angle certainly still exists. These random errors will reduce estimated heritability values compare to other traits which are less influenced by the viewing angle such as the panicle size and plant height (Table S1).

Active learning could also be employed to prioritize which pixels to select for manual annotation, rather than depending solely on user choice [9, 39, 40]. It remains an open question whether it will ultimately prove to be more effective to train separate classification models for individual crop species or whether common algorithms can be developed with application to groups of related crops. One potential approach that could be explored is transfer learning, where a model initially trained to conduct organ level classification in one species is retained when using data from a second species. In many cases, transfer learning can significantly reduce the amount of new data needed to achieve adequate performance for a new classification task [41]. However, current prediction accuracy is sufficient to enable quantitative genetic study of a range of traits (Figure 5, S5, Table S1). Therefore, the most pressing need is simply to collect image data from a larger number of genotypes, ideally with multiple replicates under a range of treatments, which would enable the identification of genes controlling variation in a range of sorghum phenotypes in diverse environments.

## Figures and Tables

**Table 1:** Cross validation accuracy for each supervised classification algorithm evaluated.

Methods	Background	Leaf	Stalk	Panicle	Average
LDA	1.000	0.969	0.946	0.974	0.972
PLS-DA	1.000	0.973	0.911	0.976	0.965
ANN	0.997	0.974	0.923	0.958	0.963
MLR	0.983	0.970	0.934	0.959	0.962
SVM	0.999	0.978	0.920	0.948	0.961
RF	0.999	0.964	0.830	0.931	0.931
LASSO	1.000	0.962	0.754	0.956	0.918
QDA	0.987	0.986	0.657	0.865	0.874

LDA: linear discriminant analysis; MLR: multinomial logistic regression; ANN: artificial neural network; SVM: support vector machine; PLS-DA: partial least squares discriminant analysis; RF: random forest; QDA: quadratic discriminant analysis; LASSO: least absolute shrinkage and selection operator.

**Table 2:** Performance of sorghum models on maize classification.

Methods	Background	Leaf	Stalk	Tassel/panicle	Average	Average (excludes tassel)
ANN	0.999	0.926	0.866	0.477	0.817	0.930
SVM	0.985	0.886	0.686	0.67	0.807	0.852
PLS-DA	0.984	0.911	0.693	0.605	0.798	0.833
LDA	1.0	0.916	0.655	0.59	0.790	0.857
RF	1.0	0.856	0.662	0.604	0.780	0.839
MLR	0.994	0.896	0.562	0.658	0.778	0.817
LASSO	0.991	0.751	0.595	0.668	0.751	0.779
QDA	0.981	0.957	0.695	0.09	0.681	0.878

## Data Availability

All the R and python code implemented in this study, phenotypes extracted from segmented sorghum images, and the manually annotated sorghum and maize pixels have been deposited on GitHub at https://github.com/freemao/Sorghum_Semantic_Segmentation. The position of each significant SNP and the nearby genes identified in the association study of panicle size, the ratio of panicle and leaf size, and the ratio of stem and leaf size were summarized in Additional file S1.xlsx.

## Supplementary Materials

Supplementary 1 Figure S1: an example of the pixel annotation interface from a Zooniverse project page. Users were asked to annotate ten pixels from each class by clicking on different positions within the image. Pixels annotated by the user as background indicated with blue crosses, leaf pixels with red crosses, stalk pixels with purple crosses, and panicle pixels with yellow crosses.

Supplementary 2 Figure S2: distribution of pixels annotated as background, leaf, stalk, or panicle in two dimensionality reduction approaches. (A) First two principal component values—as determined by principal component analysis of all annotated pixels. The proportion of total variance explained for the first and second principal components indicated in parenthesis on the *x*- and *y*-axis labels. (B) LDA1 and LDA2 values derived from linear discriminant analysis (LDA) for each annotated pixel. Both plots use the same color key for class annotations indicated in the top right corner of panel B.

Supplementary 3 Figure S3: sorghum-trained ANN confusion matrix for predictions of maize data. Based on manual annotation of a balance set of 4000 maize pixels (1000 per class). Calculated proportions are per ground truth class and sum to one in each row.

Supplementary 4 Figure S4: whole image semantic segmentation of an example maize plant using models trained on sorghum data prediction results from the five remaining sorghum models—PLS-DA, LDA, RF, MLR, and LASSO—on the same maize plant shown in Figure 4. Pixels classified as leaf, stalk, and panicle by each model are indicated in green, orange, and purple.

Supplementary 5 Figure S5: GWAS results leaf size, stalk size, panicle/leaf ratio, stalk/leaf ratio, and panicle/stalk ratio. Bonferroni corrected *P* value of 0.05 was used as the significant cutoff indicated by a horizontal dash line in each plot.

Supplementary 6 Figure S6: phenotypic differences between plants carrying the reference (A) and alternative (B) alleles for the single most significant trait-associated SNP for panicle size (SNP S10_5631741).

Supplementary 7 Figure S7: comparing the performance of the flowering models on flowering and vegetative datasets. Blue: model performance on pixels collected from plants at the vegetative development stage. Red: model performance on pixels collected from plants at the grain filling stage.

Supplementary 8 Figure S8: the distribution of pixel-based phenotypes.

Supplementary 9 Table S1: heritability and GWAS results for seven sorghum traits.

Supplementary 10 Table S2: model performance on pixels collected from sorghum plants at vegetative stage of development.

Supplementary 11 Additional file S1: the details of the significant SNPs identified by GWAS on sorghum plant height, panicle size, panicle leaf ratio, and stalk leaf ratio. The candidate genes around the significant SNPs identified by GWAS on sorghum panicle size. The candidate genes around the significant SNPs identified by GWAS on sorghum panicle leaf ratio. The candidate genes around the significant SNPs identified by GWAS on sorghum stem leaf ratio.
