# Supplementary figures and images for: Semantic Segmentation of Sorghum Using Hyperspectral Data Identifies Genetic Associations
Source: Plant Phenomics. 2020 Feb 4;2020:4216373. doi: 10.34133/2020/4216373 (PMC7706332; doi:10.34133/2020/4216373)

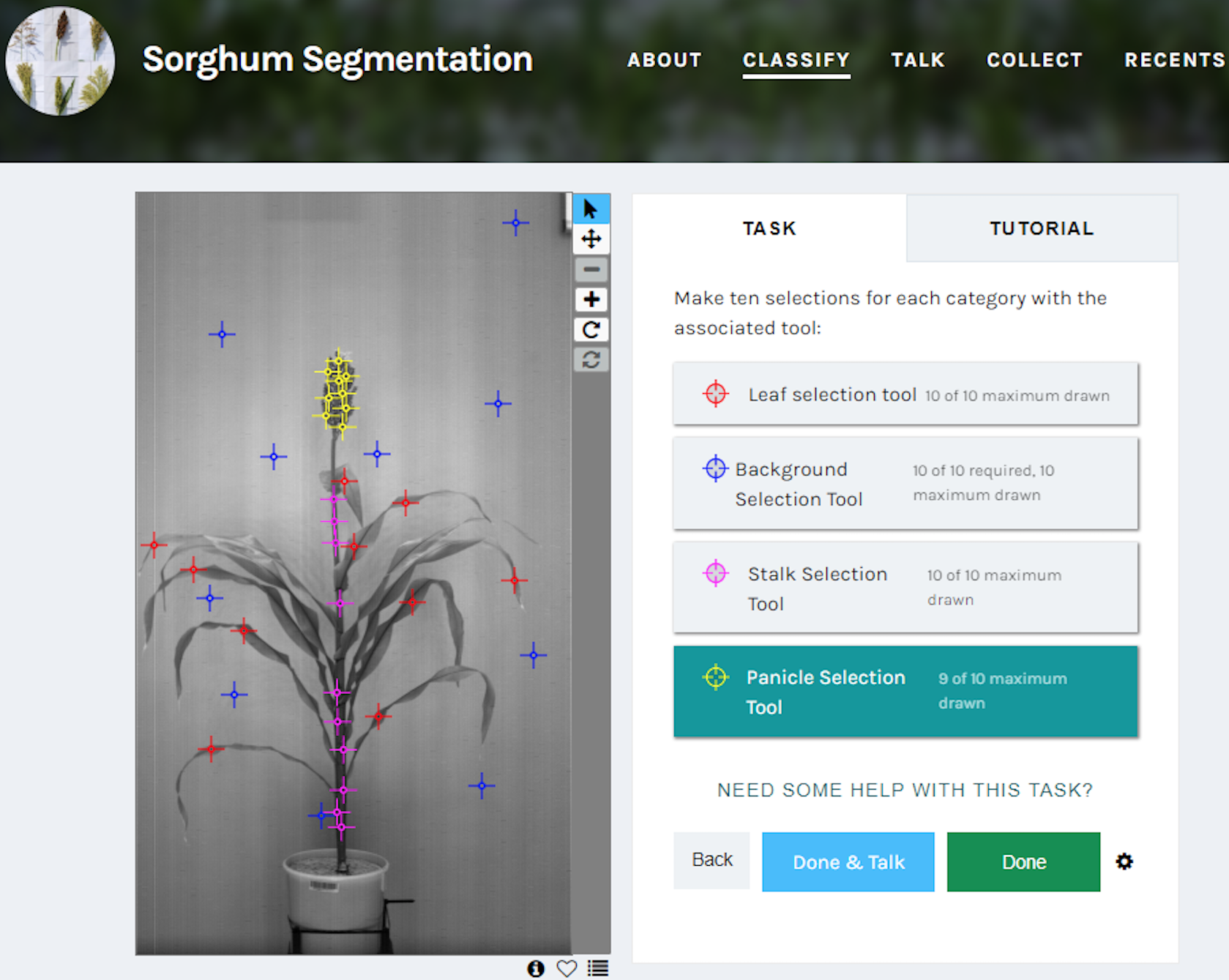

Supplement: Supplementary 1 — Figure S1: an example of the pixel annotation interface from a Zooniverse project page. Users were asked to annotate ten pixels from each class by clicking on different positions within the image. Pixels annotated by the user as background indicated with blue crosses, leaf pixels with red crosses, stalk pixels with purple crosses, and panicle pixels with yellow crosses. [file 4216373.f1.png]

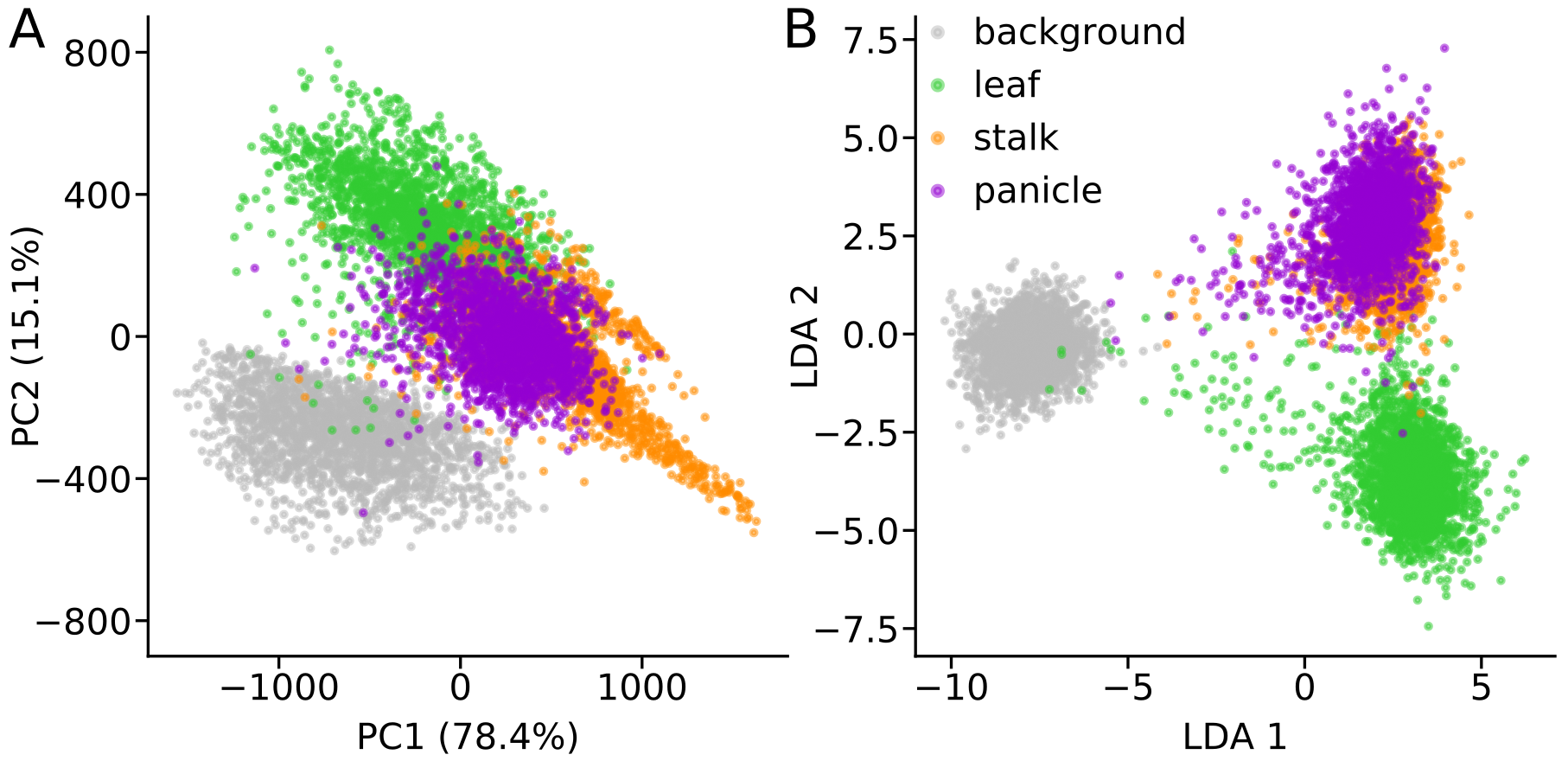

Supplement: Supplementary 2 — Figure S2: distribution of pixels annotated as background, leaf, stalk, or panicle in two dimensionality reduction approaches. (A) First two principal component values—as determined by principal component analysis of all annotated pixels. The proportion of total variance explained for the first and second principal components indicated in parenthesis on the x- and y-axis labels. (B) LDA1 and LDA2 values derived from linear discriminant analysis (LDA) for each annotated pixel. Both plots use the same color key for class annotations indicated in the top right corner of panel B. [file 4216373.f2.png]

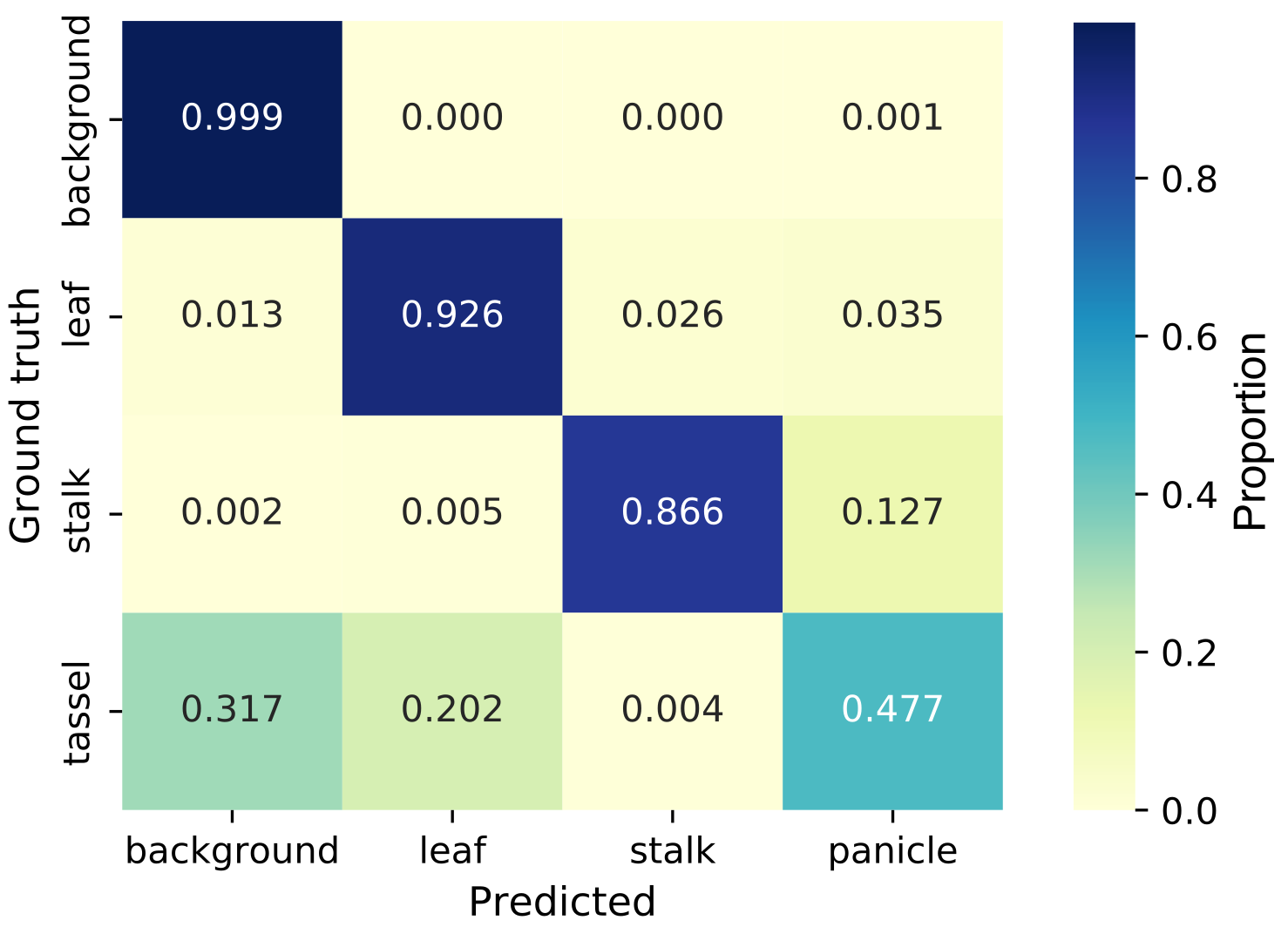

Supplement: Supplementary 3 — Figure S3: sorghum-trained ANN confusion matrix for predictions of maize data. Based on manual annotation of a balance set of 4000 maize pixels (1000 per class). Calculated proportions are per ground truth class and sum to one in each row. [file 4216373.f3.png]

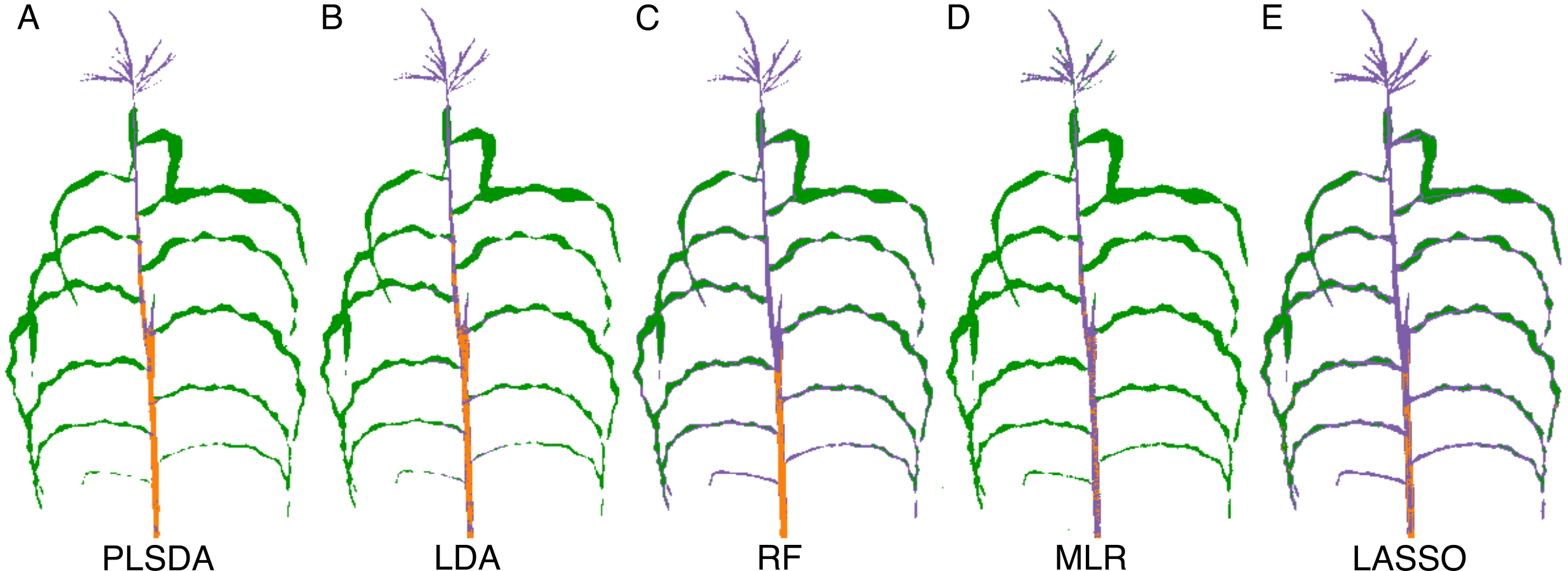

Supplement: Supplementary 4 — Figure S4: whole image semantic segmentation of an example maize plant using models trained on sorghum data prediction results from the five remaining sorghum models—PLS-DA, LDA, RF, MLR, and LASSO—on the same maize plant shown in Figure 4. Pixels classified as leaf, stalk, and panicle by each model are indicated in green, orange, and purple. [file 4216373.f4.png]

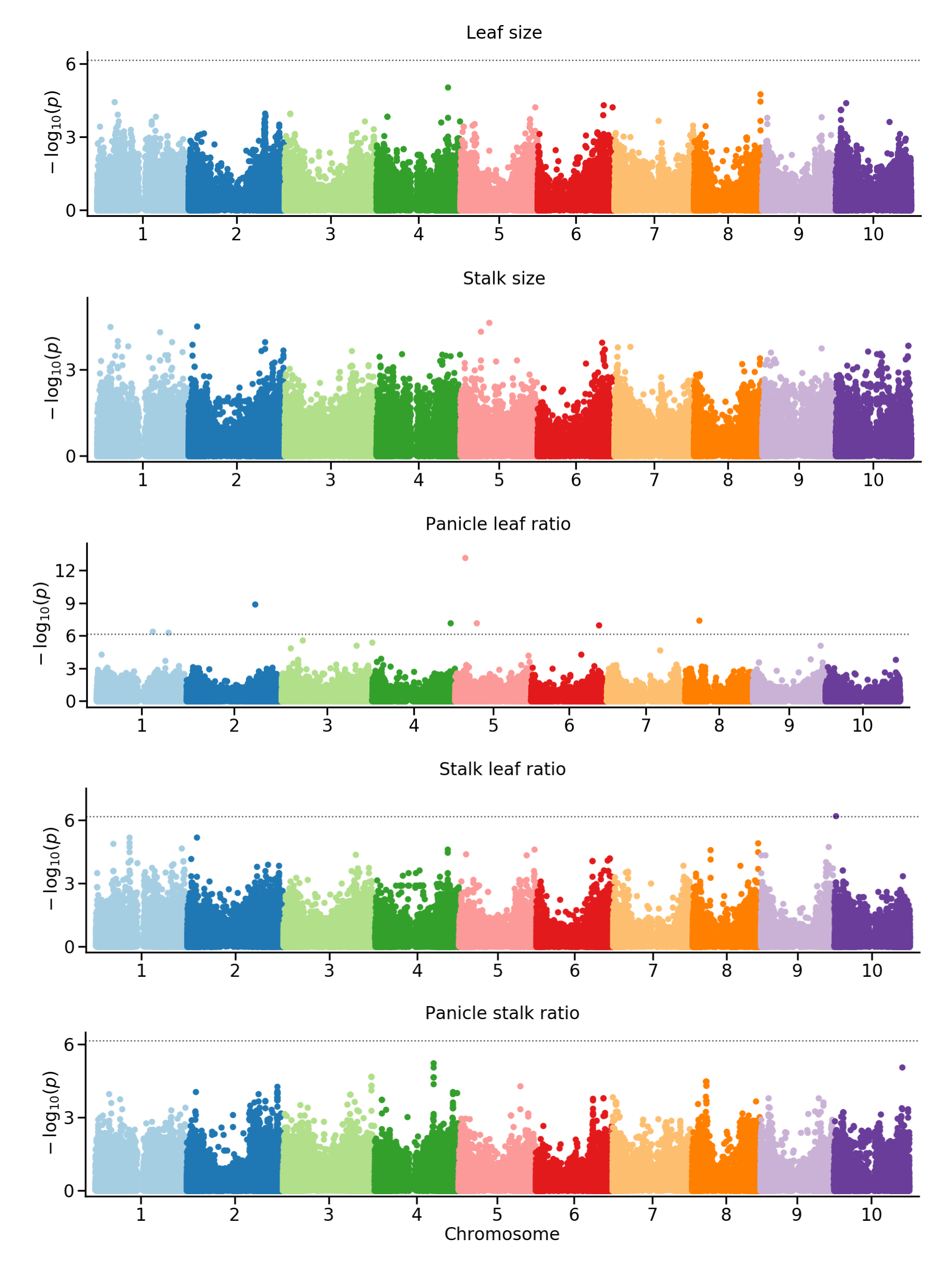

Supplement: Supplementary 5 — Figure S5: GWAS results leaf size, stalk size, panicle/leaf ratio, stalk/leaf ratio, and panicle/stalk ratio. Bonferroni corrected P value of 0.05 was used as the significant cutoff indicated by a horizontal dash line in each plot. [file 4216373.f5.png]

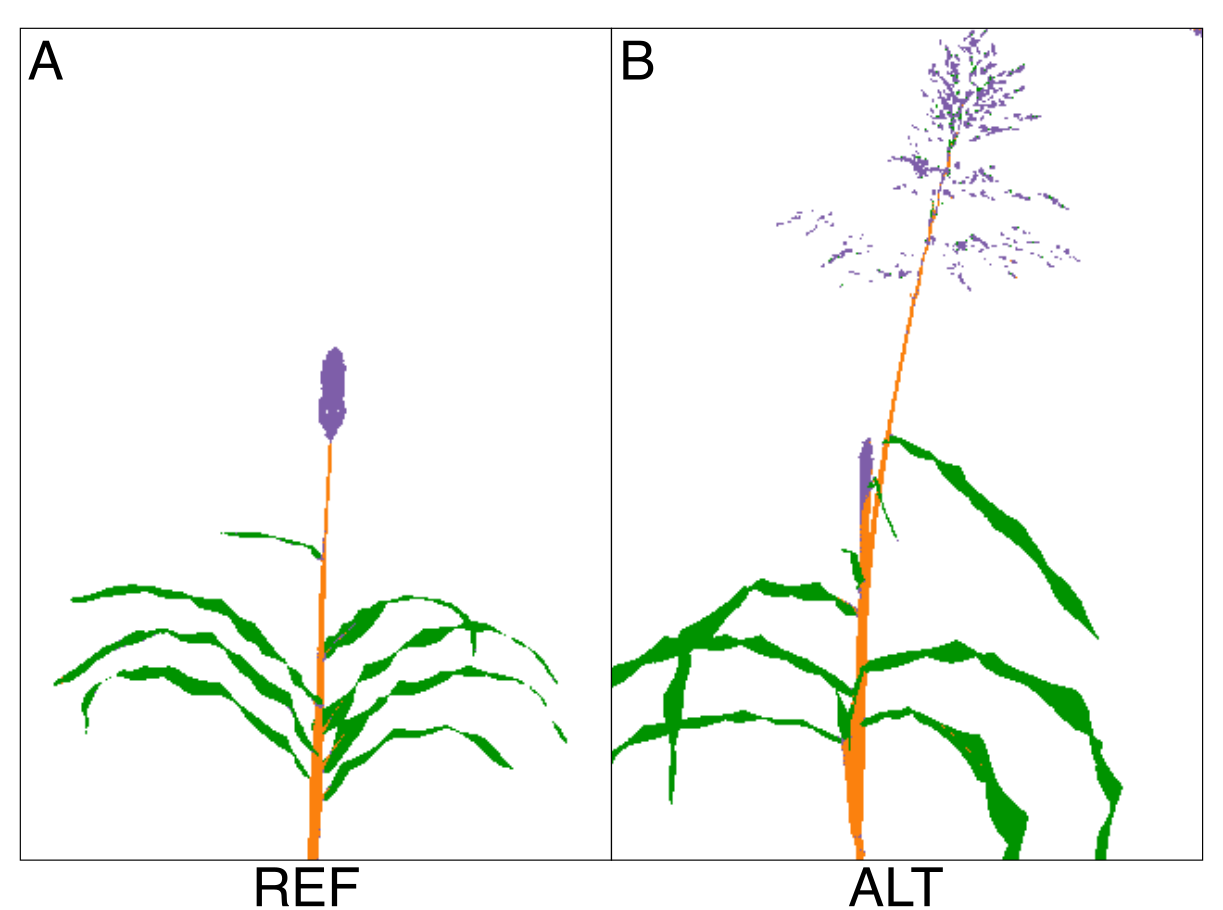

Supplement: Supplementary 6 — Figure S6: phenotypic differences between plants carrying the reference (A) and alternative (B) alleles for the single most significant trait-associated SNP for panicle size (SNP S10_5631741). [file 4216373.f6.png]

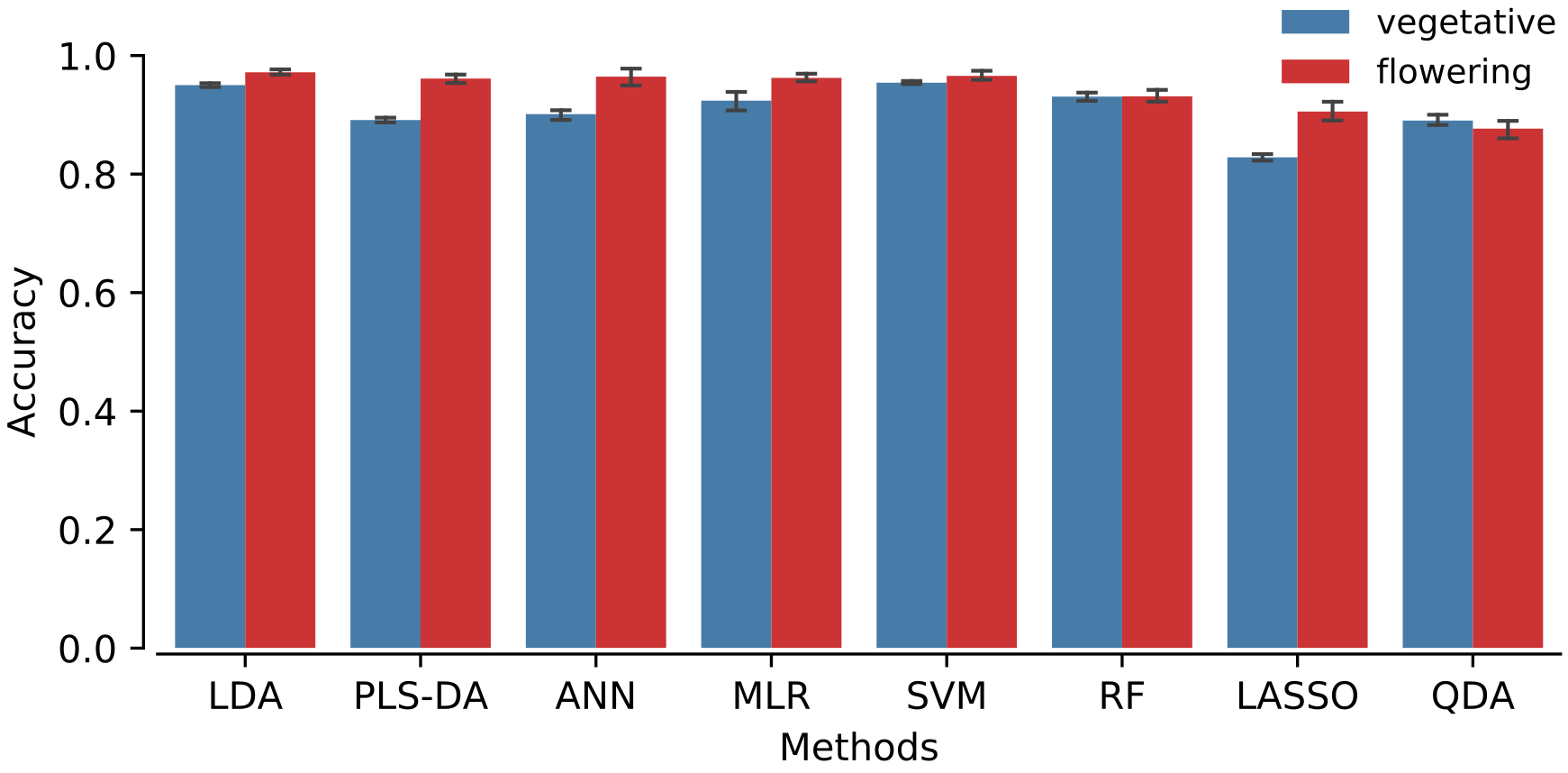

Supplement: Supplementary 7 — Figure S7: comparing the performance of the flowering models on flowering and vegetative datasets. Blue: model performance on pixels collected from plants at the vegetative development stage. Red: model performance on pixels collected from plants at the grain filling stage. [file 4216373.f7.png]

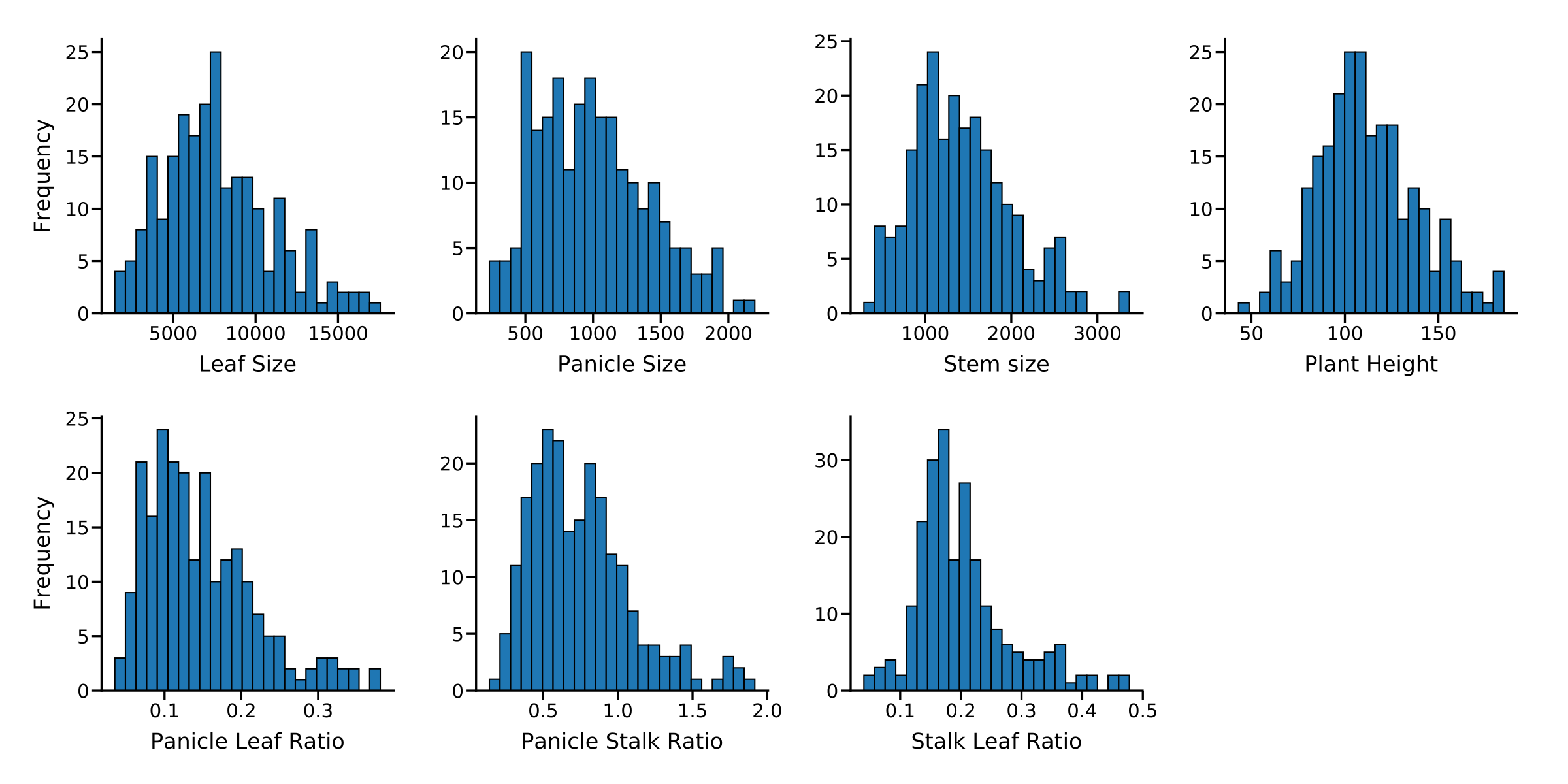

Supplement: Supplementary 8 — Figure S8: the distribution of pixel-based phenotypes. [file 4216373.f8.png]
